# The Impact of Antidepressant Therapy on Glycemic Control in Canadian Primary Care Patients With Diabetes Mellitus

**DOI:** 10.3389/fnut.2018.00047

**Published:** 2018-06-12

**Authors:** Justin Gagnon, Marie-Thérèse Lussier, Brenda MacGibbon, Stella S. Daskalopoulou, Gillian Bartlett

**Affiliations:** ^1^Department of Family Medicine, McGill University, Montreal, QC, Canada; ^2^Departement de Médecine de Famille et de Médecine d'Urgence, Université de Montréal, Montreal, QC, Canada; ^3^Department of Mathematics, Université de Québec à Montréal, Montreal, QC, Canada; ^4^Division of General Internal Medicine, Department of Medicine, McGill University, Montreal, QC, Canada

**Keywords:** diabetes mellitus, antidepressants, glycemic control, primary health care, cohort studies

## Abstract

**Context:** Depression is common in people with diabetes and is associated with poor glycemic control. Evidence suggests that certain antidepressants (AD) increase the risk of poor control. Few population-based studies have examined the impact of individual ADs on glycemic control. This study's objective is to measure the impact of Citalopram, Amitriptyline, Venlafaxine, Trazodone and Escitalopram on glycated hemoglobin (HbA1c) in Canadian primary care patients with diabetes.

**Methods:** A retrospective study of electronic medical records (EMR) from 115 primary care practices across Canada was undertaken. Data were obtained from the Canadian Primary Care Sentinel Surveillance Network (CPCSSN). The sample population comprised 1,084 diabetic patients with 1,127 prescriptions of one of the five selected ADs and with baseline and post-exposure HbA1c measurements. Generalized linear mixed models were computed to estimate the effect of the ADs on HbA1c.

**Results:** Mean HbA1c ratios for Amitriptyline, Venlafaxine, Trazodone and Escitalopram were all numerically lower than Citalopram. The confidence intervals included the minimum detectable effect, however the differences were not statistically significant. The lowest clinically relevant HbA1c ratios, relative to Citalopram, were found in patients prescribed Trazodone and Escitalopram. Accounting for the prescription of Trazodone for indications other than depression, this research suggests that Escitalopram may be safer than Citalopram for people with diabetes and depression, in terms of its effect on blood glucose.

**Conclusion:** This study can inform future research examining the relationship between ADs and blood glucose and provides insight into the limitations pertaining to the use of health data in health research. Future research should seek to control for, across multiple time points: depression symptoms, depression severity, depression duration, weight, diabetes medication, tobacco and alcohol consumption and other medications with a known impact on blood glucose.

## Introduction

Depression is a common comorbidity in people with diabetes mellitus, which increases the risk of poor health outcomes (1–4). People with diabetes and depression are at greater risk of poor diabetes control, diabetes-related complications, multimorbidity and mortality compared with those with either condition alone (4–9). The relationship between diabetes and depression is bidirectional. Depression is associated with a decline in self-management behaviors (2, 10) as well as pathophysiology linked to impaired glucose metabolism (11), and people with diabetes are at increased risk of depression (12). While treatment of depression is expected to break this cycle, evidence suggests that some antidepressant medications (AD) directly and indirectly interfere with normal glucose metabolism (13).

ADs are most often prescribed in primary care (14). The pharmacological classes of ADs most commonly prescribed in Canadian primary care are: selective serotonin reuptake inhibitors (SSRI); serotonin-norepinephrine reuptake inhibitors (SNRI), tricyclic antidepressants (TCA), serotonin antagonist reuptake inhibitors (SARI), norepinephrine-dopamine reuptake inhibitors (NDRI), noradrenergic and specific serotonergic antidepressants (NaSSA) (15). In a preliminary study of prescription practices, we found that Citalopram (SSRI), Amitriptyline (TCA), Venlafaxine (SNRI), Trazodone (SARI) and Escitalopram (SSRI) were the most frequently prescribed ADs for people with diabetes in Canada (16).

Research has linked Citalopram with improved glucose metabolism (17, 18) and weight loss (18), which can reduce the risk of poor glycemic control. The other four medications have not been studied as extensively. While the effects of Amitriptyline on glucose metabolism are inconclusive (19), it has been associated with weight gain (20), which can cause insulin resistance and poor diabetes control (21). Other TCAs (i.e., Imipramine) have been associated with impaired glucose control (22). The results of trials examining the impact of Venlafaxine are also inconclusive (20); however, Duloxetine, another SNRI, has been linked to weight loss (23). Less is known about the impact of Trazodone (SARI) on glycemic control. Escitalopram has not been studied as extensively, but other SSRIs are generally associated with improved glucose metabolism (24–26), with mixed associations of weight gain and weight loss (27). The volume of research on particular ADs is disproportional to the frequency with which they are prescribed, as more evidence exists for some of the less commonly prescribed ADs. Moreover, the findings of trials in this field are inconsistent, due in large part to the heterogeneity of study designs and sample populations.

Most observational studies have focused on the association between AD use overall, or grouped by pharmacological class, and diabetes onset. A number of epidemiological studies have reported an association between AD use in general and increased risk of diabetes onset (28–32). With regard to the pharmacological classes, SSRIs, TCAs and SNRIs have been associated with increased risk of diabetes onset, with TCAs (33) and concurrent use of SSRIs and TCAs (34) being associated with the greatest increase in risk. ADs within the same class may differ in terms of their impact on glucose metabolism (35), therefore ADs should be examined individually; however, epidemiological research has seldom examined the impact of individual ADs. Given the need for more epidemiological research in this area, the purpose of this study is to estimate the impact of Citalopram, Amitriptyline, Venlafaxine, Trazodone, and Escitalopram on glycemic control in Canadian primary care patients with diabetes.

## Methods

### Data source and study population

**Figure 1:**
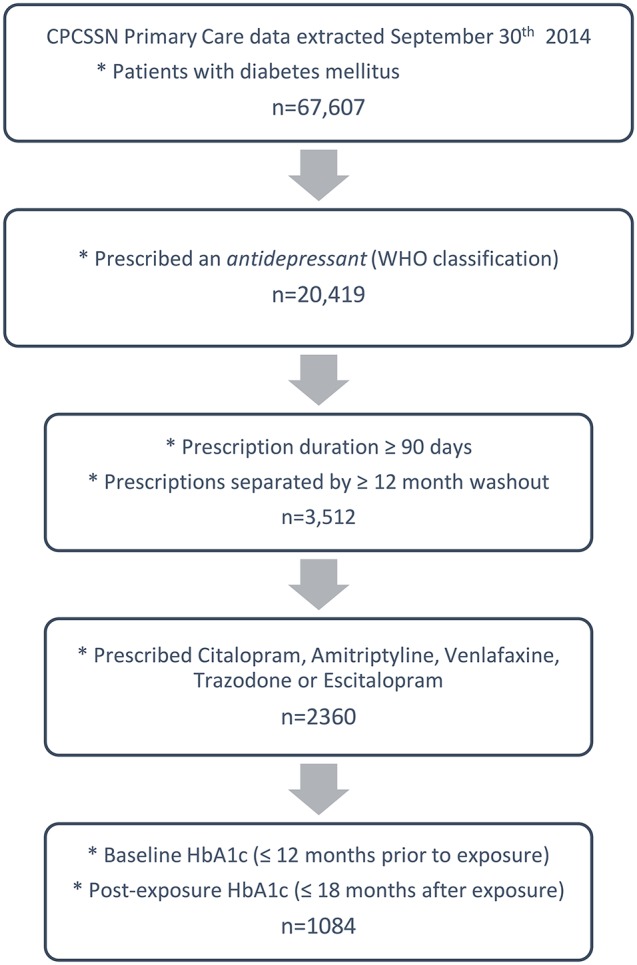
Illustration of study sample selection.

This is a retrospective cohort study of electronic medical records (EMR) from primary care providers across Canada. Data were obtained from the Canadian Primary Care Sentinel Surveillance Network (CPCSSN). The CPCSSN database was developed for chronic disease surveillance and research. The CPCSSN is somewhat representative of the general Canadian population, however older adults are over-represented and young adult males are under-represented (36). EMR data from 115 primary care practices in 9 Canadian provinces and 1 territory were extracted, anonymized, cleaned and coded by the CPCSSN in September 2014 (with data extending as far back as had been recorded electronically). Furthermore, they developed and validated disease case detection algorithms based on the combination of problem lists, medication prescriptions, billing codes and lab results to compensate for the lack of systematic and standardized entry of information in patients' electronic charts. Included in this study are diabetic patients who were prescribed either Citalopram, Amitriptyline, Venlafaxine, Trazodone, and Escitalopram; and had at least one baseline and post-exposure HbA1c measure (*n* = 1,084). Figure 1 provides an illustration of the sample selection, which is described in greater detail below.

#### Diabetes mellitus

Diabetes mellitus (identified in 66,617 patients at the moment of extraction) was identified using the validated (37) CPCSSN case detection algorithm, which identifies cases using a combination of patients' health problem list, medication prescription records, laboratory results and billing information. The case definition includes type 1 diabetes (T1DM) and type 2 diabetes (T2DM). A recent validity test obtained a positive predictive value of 87.0 (83.5–90.5), and a negative predictive value of 99.1 (95% CI: 98.6–99.6) compared to detailed chart review conducted by the primary healthcare provider (37).

#### Antidepressant medications (exposure)

Among patients with diabetes, 20,419 had a record of an AD prescription. Anatomical Therapeutic Chemical (ATC) codes were assigned to all medications in patient health records. Medications categorized as *antidepressants* by the World Health Organization (WHO) Collaborating Centre for Drug Statistics Methodology (38) were included.

Series of AD prescriptions (separated by 15 days or less) were joined to define periods of continuous use. Exclusion criteria consisted of: concurrent prescriptions of different ADs; AD prescription periods occurring within 1 year of one another (washout); and continuous prescriptions lasting less than 90 days. The 1-year washout was selected in an attempt to restrict the population to those suffering from new depressive episodes (not chronic AD users or chronic sufferers of depression), to exclude as many as possible of people prescribe the AD for other conditions such as chronic pain, sleep problems or anxiety disorders. Ninety days was selected because we will detect an impact on HbA1c after 90 days, and to exclude as much as possible short-term use of an AD for a minor or more acute episode (i.e., grief/mourning).

From the diabetic patients with distinct and continuous AD prescription periods (*n* = 3,512), the sample was further limited to those prescribed either Citalopram, Amitriptyline, Venlafaxine, Trazodone, and Escitalopram. The indication of prescription was not consistently recorded in EMRs, therefore, all AD prescriptions regardless of indication were considered. Medication dose was also not consistently recorded or entered in a standardized manner and could therefore not be reliably used.

The restriction of the sample to those with a minimum duration of AD use and for which AD prescriptions are separated by a washout period is expected to improve exchangeability of the comparison groups. To approximate the prescription of ADs for the treatment of depression, the sample is restricted to diabetic patients with depression (in sensitivity analyses), which further ensures exchangeability, and limits the inclusion of patients prescribed ADs for other indications than depression. The CPCSSN case definition for depression predicts a patients' history of having a clinical diagnosis of depression, therefore the possibility remains that some patients in the sample were prescribed an AD for another indication, but had previously suffered from depression (or the depression disease case was a false positive).

#### Glycated hemoglobin (outcome)

**Figure 2:**
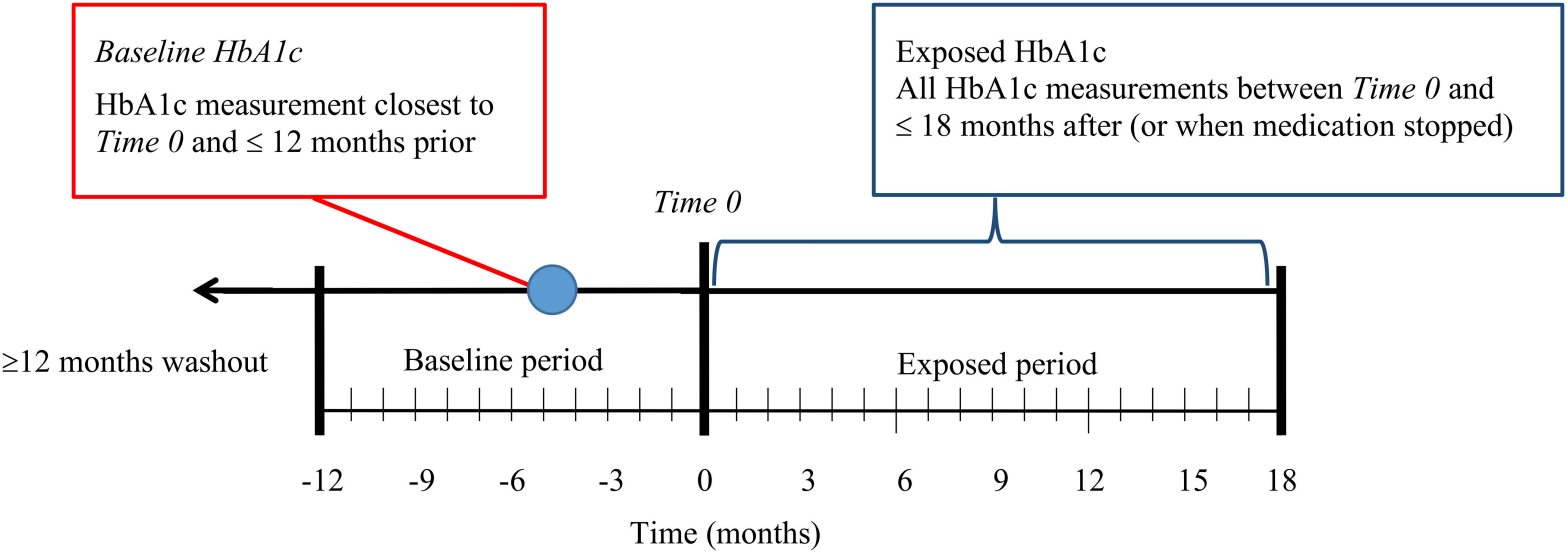
Illustration of baseline and exposed blood sugar measures.

From the patients prescribed 1 of the 5 selected ADs, those with baseline and post-exposure (post AD prescription) measures were selected. The HbA1c measurement closest to the AD prescription start date (*Time 0*) and no more than 12 months prior was used as the *baseline* value. Post-exposure HbA1c values were all HbA1c measurements taken within 18 months after *Time 0*, or until the medication was stopped. While HbA1c approximates the mean glucose concentration of the 3 previous months, the first 3 months were included to detect possible short-term effects of ADs, which were assessed using sensitivity analyses. *Exposure duration* was defined as the number of days from *Time 0* when post-exposure HbA1c was measured. Figure 2 provides an illustration the baseline and outcome measurements used.

#### Covariates

**Figure 3:**
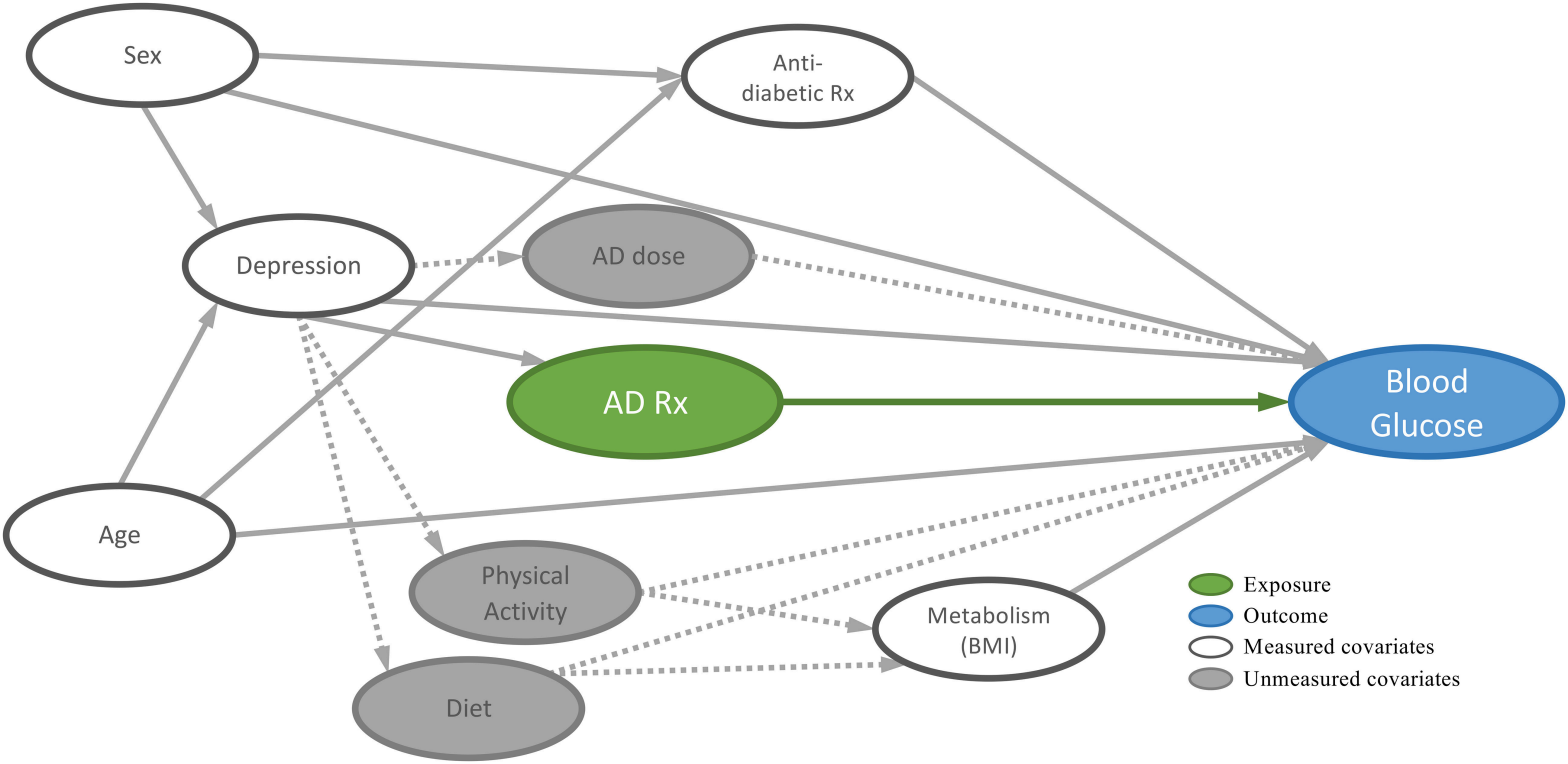
Directed acyclic graph (DAG) of the relationship between antidepressants (AD) and blood glucose.

A conceptual model (directed acyclic graph), representing the theorized relationship between ADs and blood glucose, with potential covariates, was developed according to the research literature (Figure 3). The following variables were included in the analyses: baseline HbA1c, age, sex, body mass index (BMI), diabetes medication type and depression. Age at the moment of data extraction was computed using the patients' dates of birth. A median BMI was computed for each of the patients using all recorded BMI values, when available. The median was selected because the reliability of BMI values was suspect and the median reduced the influence of potential outliers. Diabetes medication type comprised 4 categories: no medication; non-insulin medication (biguanides, sulfonylureas, sulfonamides, alpha glucosidase inhibitors, thiazolidinediones, dipeptidyl peptidase 4 inhibitors, glucagon-like peptide-1 analogs, and sodium-glucose co-transporter 2 inhibitors); combined non-insulin medication and insulin; and insulin only (39). The case definition for history of depression includes depressive disorders as well as bipolar and manic mood disorders. The definition was found to have a positive predictive value of 79.6 (95% CI: 75.7–83.6) and a negative predictive value of 95.2 (95% CI: 94.1–96.3) in a validation study that compared the CPCSSN disease case with clinical diagnoses of depression identified through detailed chart review by each patient's primary healthcare provider (37). Diet and physical activity are poorly charted and could not be included in our analyses. These may be approximated using BMI, however. Other health conditions—hypertension, osteoarthritis and chronic obstructive pulmonary disease (COPD) were statistically assessed for inclusion as covariates. While the literature does not provide evidence that these conditions are confounders or mediators of the relationship between depression and diabetes, these were included as covariates as they may have a significant impact on mobility (physical activity) and diet, which can impact glucose metabolism and blood glucose levels. The CPCSSN also developed and validated disease case definitions for these conditions (37).

### Statistical analyses

Patient characteristics, stratified by the 5 ADs, were described using frequencies and proportions and means and standard deviations, as appropriate. Baseline and post-exposure HbA1c measures were described using means and standard deviations.

A generalized linear mixed (GLM) model (40) was computed in order to estimate the impact of ADs on repeated HbA1c measures. GLM models are ideal for longitudinal data with non-normally distributed dependent variables (which is often the case with health data) as they permit modeling of random and/or fixed error terms at the level of clusters (within subjects) and the whole (between subjects) (40). Analyses were clustered at the level of individual patient-prescription periods. The logarithmic link function was used to account for the positive skew of the outcome data. The impact of ADs on HbA1c computed using the GLM model was represented in terms of mean ratios for Amitriptyline, Venlafaxine, Trazodone and Escitalopram relative to Citalopram, the most frequently prescribed of these ADs for the treatment of depression (16). Model fitness was assessed for inclusion of hypertension, osteoarthritis and COPD as covariates using the Akaike Information Criterion (AIC).

An initial model was computed which included all patients prescribed 1 of the 5 ADs. Sensitivity analyses were performed to account for BMI and history of depression. A sub-model that included only patients with BMI measurements was computed, followed by a subsequent sub-model including only those with a history of depression. Furthermore, sensitivity analyses were performed to estimate the impact of ADs for specific periods of exposure. Four additional sub-models were computed for each of the 3 models described above in which post-exposure HbA1c measurements were divided into periods of exposure: 0–3 months; 3–6 months; 6–12 months; and 12–18 months.

#### Power calculations

A total of 1,084 patients were fulfilled all the eligibility criteria and included in the analyses. Analyses were performed at the level of *patient prescription-periods* (prescriptions of longer than 90 days and separated by a 12-month washout period) of which there were 1,127. Using an *F*-test MANOVA using a sample size of 505 (5 times the smallest group – Escitalopram; *n* = 101), α of 0.05, power of 0.8 and 5 groups, the required effect size was estimated at 2.4% (using G^*^Power version 3.1). The analyses were performed using SAS version 9.4.

### Ethics

CPCSSN received ethics approval from the research ethics boards of all host Universities for all participating networks and from the Health Canada Research Ethics Boards. The present study received ethics approval from the McGill University Faculty of Medicine Research Board.

## Results

### Population characteristics

A total of 1,084 patients with 1,127 prescription-periods were included in the GLM analyses. Table 1 presents the characteristics of the population, stratified by the prescribed AD. Citalopram was most frequently prescribed (29.3%), followed by Amitriptyline (27.6%), Venlafaxine (17.4%), Trazodone (16.7%), and Escitalopram (9.0%). The groups showed clinically relevant differences with respect to age, sex, history of depression and osteoarthritis (Chi-Square). The patients given Escitalopram had a lower mean age then those prescribed the other medications, followed by Venlafaxine. A relevantly lower proportion of females were prescribed Trazodone than Venlafaxine (OR = 0.49; 95%CI = 0.32–0.74) and Citalopram (OR = 0.68; 95%CI = 0.47–0.98). Additionally, AD groups differed according to history of depression; the greatest proportion of patients with a history of depression were on Citalopram and Escitalopram (76.9 and 70.7%, respectively). No clinically relevant differences were found between AD groups for BMI, hypertension, COPD and diabetes medication type. Comparison of the characteristics of the subset of patients with a history of depression (Supplementary Table 1) shows only statistically significant differences between the AD groups according to sex and osteoarthritis. A significantly greater proportion of females were prescribed Amitriptyline compared to the other ADs, and a greater proportion of patients with osteoarthritis were prescribed Amitriptyline or Trazodone than the other ADs.

**Table 1:** Characteristics of diabetic patients prescribed Citalopram, Amitriptyline, Venlafaxine, Trazodone, or Escitalopram stratified by antidepressant agent.

	**Citalopram**	**Amitriptyline**	**Venlafaxine**	**Trazodone**	**Escitalopram**	**Total**
Total	320(29.3)	302(27.6)	190(17.4)	183(16.7)	99(9.0)	1, 094^*^(100)
**AGE (YEARS)**						
Age - mean (*sd*)	67.6(13.8)	67.5(11.1)	64.7(12.2)	69.8(14.1)	61.9(14.4)	67(13.1)
**SEX**						
Male	134(41.9)	130(43.0)	65(34.2)	94(51.4)	42(42.4)	465(42.5)
Female	186(58.1)	172(57.0)	125(65.8)	89(48.6)	57(57.6)	629(57.5)
**BMI**						
Underweight (< 18.5)	1(0.5)	0(0)	0(0)	0(0)	0(0)	1(0.1)
Normal (18.5–24.9)	28(12.7)	28(12.6)	17(12.4)	20(15)	7(9.3)	100(12.7)
Overweight (25–29.9)	61(27.7)	55(24.8)	36(26.3)	40(30.1)	22(29.3)	214(27.2)
Obese (≥30)	130(59.1)	139(62.6)	84(61.3)	73(54.9)	46(61.3)	472(60)
**HEALTH CONDITIONS**						
Depression	246(76.9)	59(19.5)	106(55.8)	57(31.1)	70(70.7)	538(49.6)
Hypertension	217(67.8)	199(65.9)	128(67.4)	124(67.8)	58(67.8)	726(66.4)
Osteoarthritis	89(27.8)	115(38.1)	49(25.8)	79(43.2)	39(39.4)	371(33.9)
COPD	58(18.1)	50(18.1)	20(10.5)	29(18.9)	10(10.1)	167(15.3)
**ANTIDIABETIC RX**						
Insulin and Non-insulin	85(26.6)	89(29.5)	52(27.4)	47(25.7)	20(20.2)	293(26.8)
Insulin only	17(5.3)	23(7.6)	17(8.9)	10(5.5)	11(11.1)	78(7.1)
Non-insulin only	168(52.5)	145(48)	95(50)	97(53)	49(49.5)	554(50.6)
No diabetes Rx	50(15.6)	45(14.9)	26(13.7)	29(15.8)	19(19.2)	169(15.4)

* *10 patients were included in more than one column as they were prescribed different antidepressants on separate occasions. The means and proportions in the Total column were computed for the 1,084 patients*.

### Impact of antidepressants on HbA1C

Table 2 provides a comparison of mean change in HbA1c following AD exposure from baseline for patients prescribed Citalopram, Amitriptyline, Venlafaxine, Trazodone or Escitalopram. At baseline, HbA1c values of patients prescribed Citalopram were more elevated than those prescribed Venlafaxine (mean difference = 0.26; 95%CI = 0.02–0.49) and Trazodone (mean difference = 0.39; 95%CI = 0.15–0.63). No statistically significant differences in post-exposure HbA1c change, relative to baseline, were found between ADs. The largest decrease in HbA1c post-exposure was observed between 3 and 6 months following AD exposure in the group prescribed Escitalopram (−0.29; *sd* = 0.90); and the largest increases were observed between 6 and 12 months in the group prescribed Amitriptyline (0.21; *sd* = 1.28), and between 12 and 18 months in the group prescribed Venlafaxine (0.21; *sd* = 0.91).

**Table 2:** Mean change in HbA1c from baseline stratified by antidepressant agent.

	**Citalopram**	**Amitriptyline**	**Venlafaxine**	**Trazodone**	**Escitalopram**	**Total**
	***n* = 333**	***n* = 312**	***n* = 195**	***n* = 186**	***n* = 101**	***n* = 1,127**
	**mean (*sd*)**	**mean (*sd*)**	**mean (*sd*)**	**mean (*sd*)**	**mean (*sd*)**	**mean (*sd*)**
Baseline HbA1c	7.4(1.5)	7.2(1.4)	7.1(1.3)	7.2(1.4)	7.4(1.9)	7.2(1.5)
**CHANGE IN HbA1c FROM BASELINE**						
Total	−0.01(1.5)	0.06(1.14)	0.06(0.95)	−0.07(0.97)	−0.17(0.87)	0.001(1.08)
0–3 months	−0.16(0.73)	−0.05(1.05)	−0.05(0.73)	−0.19(0.93)	−0.25(0.92)	−0.12(0.88)
3–6 months	−0.21(0.87)	−0.03(0.97)	−0.04(1.03)	−0.08(1.03)	−0.29(0.90)	−0.11(0.96)
6–12 months	0.14(1.51)	0.21(1.28)	0.14(1.07)	0.03(1.03)	−0.08(0.82)	0.13(1.13)
12–18 months	0.19(1.34)	0.09(1.20)	0.21(0.91)	0.01(0.80)	0.06(0.77)	0.13(1.13)

**Figure 4:**
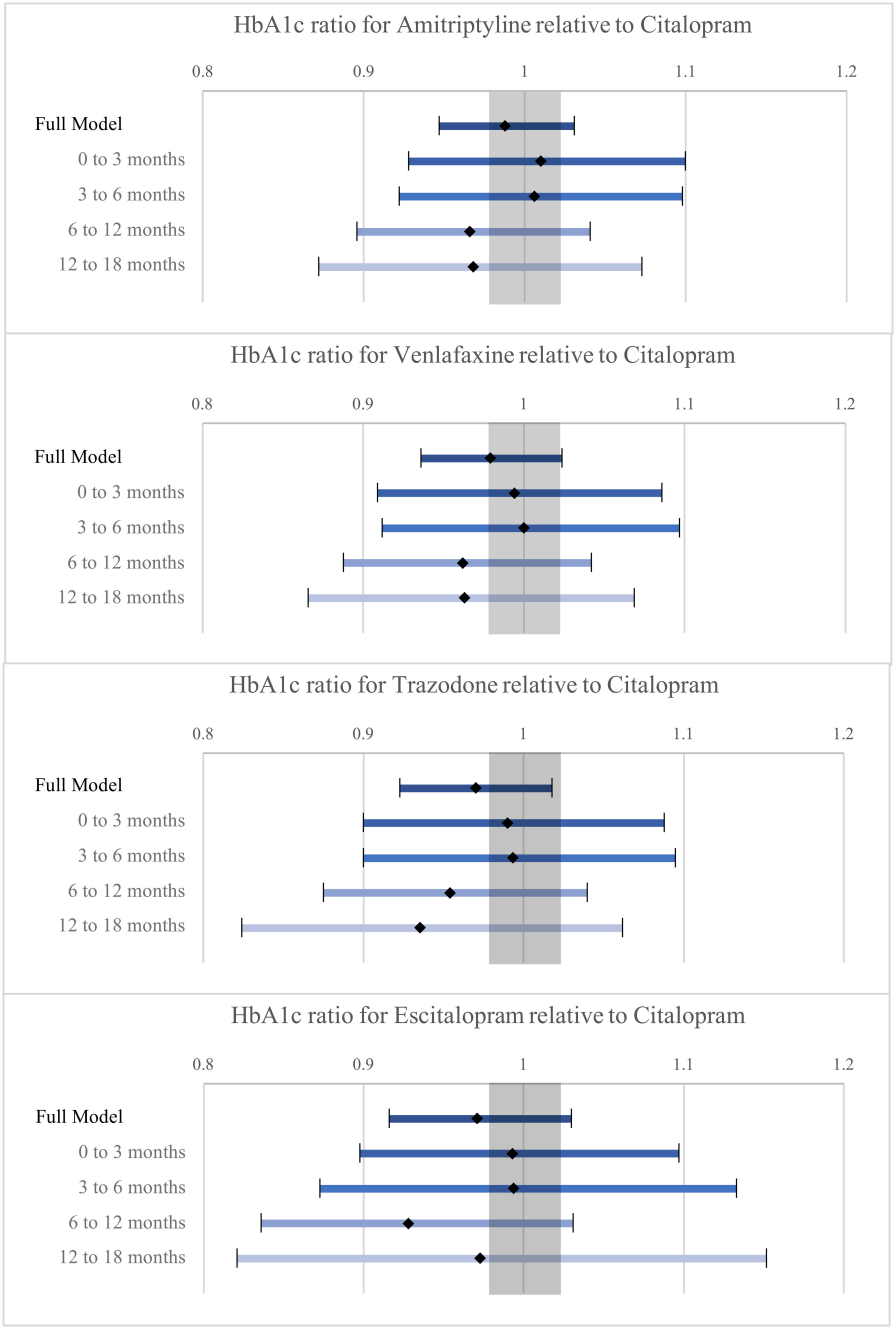
Illustration of the Mean HbA1c ratio estimates of Amitriptyline, Venalafaxine, Trazodone and Escitalopram relative to Citalopram with 95% confidence intervals (shaded area corresponds to minimum detectable effect size).

The results of the GLM model for the full sample population (*n* = 1,127) are presented in Table 3 and displayed in Figure 4 (the complete table is presented in Supplementary Table 2). The figure also presents the results of sub-models computed for specific periods of exposure to the ADs (0–3 months; 3–6 months; 6–12 months; and 12–18 months). The table shows that mean HbA1c was lower for the four ADs compared to Citalopram (as the mean ratios were all less than 1.00). The 95% confidence intervals all crossed the line of unity and were thus consistent with a possible null effect (no difference), however the confidence intervals all included the minimum detectable effect (2.4%). Trazodone had the lowest proportion relative to Citalopram at 97.0%. The sensitivity analyses examining the impact of ADs on HbA1c for different periods of exposure showed that between 6 and 12 months after AD exposure, Trazodone and Escitalopram had the lowest proportional means compared to Citalopram. However, none of these comparisons were statistically significant.

**Table 3:** Model predicting the association between antidepressants and mean HbA1c ratio in people with diabetes (*n* = 1,127).

	**Mean HbA1c ratio**	**95% CI**
Baseline HbA1c	1.079	1.068–1.091
**ANTIDEPRESSANTS**		
**Citalopram**	**(ref)**	**(ref)**
Amitriptyline	0.988	0.947–1.031
Venlafaxine	0.979	0.936–1.024
Trazodone	0.970	0.923–1.018
Escitalopram	0.971	0.916–1.030
Exposure duration (days)	1.000	1.000–1.000
**CHARACTERISTICS**		
Age	0.999	0.998–1.000
Sex (female)	1.015	0.985–1.045
History of depression	0.976	0.943–1.010
**ANTIDIABETIC MEDICATION TYPE**		
**No diabetes medication**	**(ref)**	**(ref)**
Insulin and Non-insulin	1.075	1.020–1.133
Insulin only	1.093	1.022–1.168
Non-insulin only	1.010	0.962–1.060

BMI was not included in the initial model as this variable contained a number of missing values (27.4% of patients had no BMI measurements). Supplementary Table 3 provides the results of the model that includes adjustment for BMI using the subset of patients with BMI measurements (*n* = 811). Like the previous model, mean HbA1c was lower for the 4 ADs compared to Citalopram. In the sub-models which distinguished different periods of exposure, the mean HbA1c ratios were higher for all ADs compared to Citalopram between 3 and 6 months of exposure. The highest proportional mean was observed in the group prescribed Amitriptyline (102.2%). After 6 months of exposure, the mean HbA1c ratios for all ADs were lower than Citalopram. Again, as in the previous model, the lowest mean ratios were observed for Escitalopram and Trazodone after 6 months of exposure.

Supplementary Table 4 provides the results of the model computed using patients with diabetes, a history of depression and BMI measurements (*n* = 404). The results of this model show relatively comparable mean HbA1c ratios for the 4 ADs. The confidence intervals all crossed the line of unity and were relatively wide compared to the previous two tables (as is consistent with smaller sample sizes). As with the previous tables, the smallest mean ratios were observed for the group prescribed Escitalopram and Trazodone after 6 months.

With regard to the covariates, use of insulin and combined non-insulin medication and insulin were associated with a statistically significant increase in mean HbA1c relative to no diabetes medication, whereby the confidence intervals included the line of unity as well as the minimum detectable effect. In those with a history of depression, the mean HbA1c was lower than those without, with only a slight intersection of the confidence intervals with the line of unity. The mean HbA1c for those with a history of depression was lower than those without, however the associated confidence intervals were consistent with clinically irrelevant differences. The data did not provide evidence that age and duration of exposure are relevantly associated with HbA1c.

## Discussion

This study estimated the effect of Amitriptyline, Venlafaxine, Trazodone, and Escitalopram on HbA1c compared to Citalopram for patients with DM using a large Canadian primary care EMR database. Although statistically significant differences in HbA1c could not be detected, as the confidence intervals for the mean HbA1c ratios crossed the line of unity, the confidence intervals included the minimum detectable effect. While the null hypothesis could not be rejected, the possibility of Amitriptyline, Venlafaxine, Trazodone, and Escitalopram being associated with lower mean HbA1c values than Citalopram is not dismissed. Sensitivity analyses comparing periods of exposure (>6 months) find that Trazodone and Escitalopram consistently showed the largest relative difference in HbA1c compared to Citalopram. Lower mean HbA1c ratios for Trazodone and Escitalopram were also observed in the sub-model controlling for BMI as well as the model computed for the subset of patients with a history of depression.

These findings, while not statistically significant, suggest that Trazodone and Escitalopram may be associated with clinically relevant lower blood glucose (hypoglycemic effect or reduced hyperglycemic effect) than Citalopram, the standard of care for people with depression, if we can assume that those prescribed the different ADs are exchangeable. The reduction of the sample to people with a history of depression (Supplementary Table 1) led to more comparable characteristics between the groups, with Amitriptyline and Trazodone having more questionable exchangeability. It is known that Amitriptyline and Trazodone are commonly prescribed for other conditions than depression (Amitriptyline for chronic pain and Trazodone for insomnia, both of which may be related to osteoarthritis, which was more prevalent among these) (41). Results for these medications should therefore be interpreted with caution. We have confidence in the exchangeability, however, between those prescribed Escitalopram and Citalopram. Our finding a clinically relevant, lower mean HbA1c in those prescribed Escitalopram compared to Citalopram, therefore, suggests that Escitalopram may be safer in terms of its impact on blood glucose. Research on the mechanisms linking ADs and blood glucose supports our findings (35, 42). Future research should explore these findings in a larger sample of patients specifically prescribed these ADs for the treatment of depression, and whose course for both DM and depression are comparable or controlled for (in terms of DM and depressive episode duration, changes in anti-diabetic medications and depression severity).

Mean HbA1c ratios at baseline, as well as ratios within the first 6 months of AD exposure were relatively comparable. As HbA1c is a measure that estimates the average glucose concentration of the previous 90–120 days (43), observation of the metabolic effect of ADs was not expected within this time frame. The lower mean HbA1c ratio observed for patients with a history of depression was contrary to what was expected, given that depression is generally associated with poorer glycemic control (4). This finding might suggest closer monitoring among patients with depression, which is consistent with the literature (44).

### Limitations

This study had a number of important limitations, specifically regarding the content and quality of the dataset. The CPCSSN database consists of medical data, entered by healthcare providers for clinical purposes. While this real-world medical data is extremely valuable for observational research, the data were derived from multiple healthcare providers using diverse EMR products. While the lack of standardization of EMR fields and data entry can affect the availability and reliability of the data, the CPCSSN has performed a great amount of cleaning and coding, which provides standardization and vastly improves the reliability of the medical data for use in research. What remains an issue, however, are fields that are not consistently used by healthcare providers and fields that have not yet been coded sufficiently. Other studies have recommended including the following variables, which could not be included in this study: diet, physical activity, smoking status, alcohol consumption, dyslipidemia, referral to a psychotherapist or combined cognitive and pharmacological depression treatment, indication for AD prescription, severity of depression and AD dose. AD dose, especially, would have permitted an estimation of a dose-response relationship between the ADs and HbA1c, which has been observed in other research (45). The HbA1c estimates may have been mediated by AD dose, which is linked to the indication for which the AD was prescribed (46).

Mixed effects modeling accounts for a certain degree of within-subject variation over time, as well as between-subject variation, accounting partly for unmeasured covariates. However, as patients may have been prescribed ADs for other indications than depression, use of *history of depression* (to approximate patients actively suffering from depression) over-estimates those prescribed ADs for the treatment of depression. To reduce the differences between the comparison groups, we performed sensitivity analyses on the subset of patients with a history of depression. In terms of symptoms and severity of depression, the groups may not be entirely exchangeable. Inclusion of indication for AD prescription and severity of depression could have accounted for differences in illness between patients. In addition, the differences in HbA1c observed may be attributable, in part, to mediating factors, such as loss of appetite, weight loss, or a reduction in physical activity. The impact of mediators such as these could not be assessed, as this data was not available. Therefore, our results include the direct (metabolic) and indirect (behavioral) impact of the different ADs on blood glucose.

In addition, due to inconsistent availability of dose information, changes in diabetes medications also was not included as a covariate. As diabetes medications may have been adjusted to counter increases in glucose levels resulting from depression or AD use, the hyperglycemic effects of certain ADs may be underestimated. Finally, time varying weight (or BMI) were not included as factors since the dataset contained a number of potentially erroneous BMI values (or weight and/or height were also missing or potentially erroneous), which affected the reliability of all values. As healthcare providers may be more likely weigh patients with extensive health problems and/or excessive weight, the missing values were considered non-random. Consequently, and given the unavailability of variables that might have been used in the prediction of missing information, multiple imputation for the missing values was not employed.

Another limitation is that the findings may not be generalizable to all patients with diabetes prescribed ADs, given the over-representation of older adults and under-representation of young adult men in the CPCSSN population (36). The CPCSSN data are obtained from primary care practices participating in the project. Participating primary care providers are slightly more likely to be those interested in chronic disease surveillance and use of EMRs. Despite limited generalizability to the Canadian population or to all Canadian primary care practices, the internal validity is not compromised.

This research makes a meaningful methodological contribution, in its analysis of longitudinal health data using mixed models. The use of mixed effects models is ideal for clinical data as it accounts for within- and between-subject variation over time. Future research in which exchangeability of comparison groups cannot be assumed should consider employing marginal models (40) to account for different distributions of sample characteristics that are suspected to have an impact on the exposure or outcome. Given the limited availability of demographic information and other covariates related to the exposure and outcome; this technique was not employed in this research.

Evidence on the impact of ADs and knowledge about the mechanisms linking certain ADs with impaired glucose metabolism is currently inconclusive. The results of observational studies also often fail to corroborate the findings of clinical trials (13, 47–49). In addition, studies in this field are relatively heterogeneous in terms of population and study design, making their synthesis in meta-analyses difficult. The present study is one of few cohort studies using clinical data and examining the impact of individual ADs on HbA1c. Moreover, given that the studies looking at the relationship between ADs and blood glucose to date were primarily small, selective RCTs or cohort studies with broader definitions of exposure and outcome; the present paper advances the research in this field. Furthermore, our research highlights some of the limitations pertaining to the use of health data in research and outlines directions for future research.

## Author contributions

JG was primary investigator and conducted the research under the supervision of M-TL and GB. All authors contributed on the methods and interpretation of results. The text was written by JG and revised by the other authors.

### Conflict of interest statement

The authors declare that the research was conducted in the absence of any commercial or financial relationships that could be construed as a potential conflict of interest.
