# Supplementary material for: The Impact of Antidepressant Therapy on Glycemic Control in Canadian Primary Care Patients With Diabetes Mellitus
Source: Front Nutr. 2018 Jun 12;5:47. doi: 10.3389/fnut.2018.00047 (PMC6005871; doi:10.3389/fnut.2018.00047)
Supplement: Supplementary file 1 [file Table_1.pdf]

SUPPLEMENTARY TABLE 1 | Characteristics of diabetic patients with a history of depression prescribed Citalopram, Amitriptyline, Venlafaxine, Trazodone or Escitalopram stratified by antidepressant agent (n=538).

	Citalopram	Amitriptyline	Venlafaxine	Trazodone	Escitalopram
Total	246 (45.7)	59 (11.0)	106 (19.7)	57 (10.6)	70 (13.0)
**AGE (YEARS)**					
Age - mean(sd)	67.0 (13.9)	67.5 (10.1)	65.3 (11.8)	65.0 (15.5)	61.9 (14.2)
**SEX**					
Male	108 (43.9)	17 (28.8)	39 (36.8)	22 (38.6)	26 (37.1)
Female	138 (56.1)	42 (71.2)	67 (63.2)	35 (61.4)	44 (62.9)
**BMI**					
Underweight (<18.5)	0 (0)	0 (0)	0 (0)	0 (0)	0 (0)
Normal (18.5-24.9)	18 (10.9)	7 (13.2)	11 (13.9)	3 (7.1)	7 (12.5)
Overweight (25-29.9)	45 (27.3)	13 (24.5)	22 (27.9)	8 (19.1)	17 (30.4)
Obese (>=30)	102 (61.8)	33 (62.3)	46 (58.2)	31 (73.8)	32 (57.1)
**HEALTH CONDITIONS**					
Hypertension	160 (65.0)	39 (66.1)	76 (71.7)	38 (66.7)	42 (60.0)
Osteoarthritis	69 (28.1)	30 (50.9)	27 (25.5)	27 (47.4)	28 (40.0)
COPD	45 (45.7)	14 (23.7)	13 (12.3)	10 (17.5)	6 (8.6)
**ANTIDIABETIC RX**					
Insulin and Non-insulin	72 (29.3)	15 (25.4)	32 (30.2)	15 (26.3)	16 (22.9)
Insulin only	14 (5.7)	5 (8.5)	8 (7.5)	2 (3.5)	10 (14.3)
Non-insulin only	122 (49.6)	32 (54.2)	51 (48.1)	29 (50.9)	33 (47.1)
No diabetes Rx	38 (15.5)	7 (11.9)	15 (14.2)	11 (19.3)	11 (15.7)

Gagnon J, Lussier M-T, MacGibbon B, Daskalopoulou SS and Bartlett G (2018) The Impact of Antidepressant Therapy on Glycemic Control in Canadian Primary Care Patients With Diabetes Mellitus. Front. Nutr. 5:47. doi: 10.3389/fnut.2018.00047
