# Supplementary material for: The Impact of Antidepressant Therapy on Glycemic Control in Canadian Primary Care Patients With Diabetes Mellitus
Source: Front Nutr. 2018 Jun 12;5:47. doi: 10.3389/fnut.2018.00047 (PMC6005871; doi:10.3389/fnut.2018.00047)
Supplement: Supplementary file 2 [file Table_2.pdf]

SUPPLEMENTARY TABLE 2 | Model predicting the association between antidepressants and mean HbA1c ratio in people with diabetes.

	Full model n=1127		0 to 3 months n=624		3 to 6 months n=551		6 to 12 months n=546		12 to 18 months n=309	
	**Mean HbA1c ratio**	**95% CI**	**Mean HbA1c ratio**	**95% CI**	**Mean HbA1c ratio**	**95% CI**	**Mean HbA1c ratio**	**95% CI**	**Mean HbA1c ratio**	**95% CI**
Baseline HbA1c	1.079	1.068 to 1.091	1.090	1.071 to 1.109	1.082	1.056 to 1.109	1.067	1.046 to 1.089	1.069	1.039 to 1.101
**ANTIDEPRESSANTS**										
**Citalopram**	**(ref)**	**(ref)**	**(ref)**	**(ref)**	**(ref)**	**(ref)**	**(ref)**	**(ref)**	**(ref)**	**(ref)**
Amitriptyline	0.988	0.947 to 1.031	1.010	0.928 to 1.100	1.006	0.922 to 1.098	0.966	0.896 to 1.041	0.968	0.872 to 1.073
Venlafaxine	0.979	0.936 to 1.024	0.994	0.909 to 1.086	1.000	0.912 to 1.097	0.962	0.888 to 1.042	0.963	0.866 to 1.069
Trazodone	0.970	0.923 to 1.018	0.990	0.900 to 1.088	0.993	0.900 to 1.095	0.954	0.875 to 1.040	0.935	0.824 to 1.062
Escitalopram	0.971	0.916 to 1.030	0.993	0.898 to 1.097	0.994	0.873 to 1.133	0.928	0.836 to 1.031	0.973	0.821 to 1.152
Exposure duration (days)	1.000	1.000 to 1.000	1.000	0.999 to 1.001	1.000	0.999 to 1.001	1.000	0.999 to 1.000	1.000	0.999 to 1.001
**CHARACTERISTICS**										
Age	0.999	0.998 to 1.000	1.000	0.997 to 1.002	0.999	0.997 to 1.001	0.998	0.996 to 1.000	0.999	0.996 to 1.002
Sex (female)	1.015	0.985 to 1.045	1.014	0.958 to 1.074	1.012	0.952 to 1.075	1.023	0.971 to 1.079	1.004	0.930 to 1.084
History of depression	0.976	0.943 to 1.010	1.005	0.941 to 1.072	0.975	0.909 to 1.046	0.954	0.899 to 1.013	0.966	0.886 to 1.053
**ANTIDIABETIC MEDICATION TYPE**										
**No diabetes medication**	**(ref)**	**(ref)**	**(ref)**	**(ref)**	**(ref)**	**(ref)**	**(ref)**	**(ref)**	**(ref)**	**(ref)**
Insulin and non-insulin	1.075	1.020 to 1.133	1.035	0.939 to 1.141	1.067	0.960 to 1.187	1.108	1.004 to 1.223	1.094	0.957 to 1.249
Insulin only	1.093	1.022 to 1.168	1.042	0.916 to 1.186	1.099	0.964 to 1.255	1.118	0.989 to 1.263	1.124	0.952 to 1.327
Non-insulin only	1.010	0.962 to 1.060	0.994	0.91 to 1.087	1.016	0.923 to 1.118	1.007	0.920 to 1.103	1.024	0.904 to 1.160

Gagnon J, Lussier M-T, MacGibbon B, Daskalopoulou SS and Bartlett G (2018) The Impact of Antidepressant Therapy on Glycemic Control in Canadian Primary Care Patients With Diabetes Mellitus. Front. Nutr. 5:47. doi: 10.3389/fnut.2018.00047
