# Supplementary material for: The Impact of Antidepressant Therapy on Glycemic Control in Canadian Primary Care Patients With Diabetes Mellitus
Source: Front Nutr. 2018 Jun 12;5:47. doi: 10.3389/fnut.2018.00047 (PMC6005871; doi:10.3389/fnut.2018.00047)
Supplement: Supplementary file 3 [file Table_3.pdf]

SUPPLEMENTARY TABLE 3 | Model predicting the association between antidepressants and change in HbA1c in diabetics, adjusting for body mass index.

	Full model n=811		0 to 3 months n=445		3 to 6 months n=406		6 to 12 months n=398		12 to 18 months n=232	
	**Mean HbA1c ratio**	**95% CI**	**Mean HbA1c ratio**	**95% CI**	**Mean HbA1c ratio**	**95% CI**	**Mean HbA1c ratio**	**95% CI**	**Mean HbA1c ratio**	**95% CI**
Baseline HbA1c	1.080	1.066 to 1.093	1.093	1.07 to 1.116	1.080	1.05 to 1.111	1.069	1.045 to 1.094	1.069	1.033 to 1.105
**ANTIDEPRESSANTS**										
**Citalopram**	**(ref)**	**(ref)**	**(ref)**	**(ref)**	**(ref)**	**(ref)**	**(ref)**	**(ref)**	**(ref)**	**(ref)**
Amitriptyline	0.986	0.939 to 1.035	1.015	0.919 to 1.121	1.022	0.925 to 1.130	0.958	0.88 to 1.043	0.951	0.849 to 1.065
Venlafaxine	0.984	0.933 to 1.038	1.004	0.902 to 1.117	1.005	0.903 to 1.118	0.958	0.87 to 1.056	0.981	0.867 to 1.11
Trazodone	0.971	0.918 to 1.027	0.995	0.891 to 1.111	1.016	0.906 to 1.139	0.948	0.861 to 1.044	0.920	0.797 to 1.062
Escitalopram	0.975	0.909 to 1.046	0.995	0.883 to 1.121	1.018	0.868 to 1.193	0.942	0.829 to 1.07	0.944	0.769 to 1.158
Exposure duration (days)	1.000	1.000 to 1.000	1.000	0.999 to 1.001	1.000	0.998 to 1.001	1.000	0.999 to 1.001	1.000	0.999 to 1.001
**CHARACTERISTICS**										
Age	0.999	0.997 to 1.000	0.999	0.997 to 1.002	0.999	0.996 to 1.002	0.998	0.995 to 1.000	0.998	0.995 to 1.002
Sex (Women)	1.009	0.975 to 1.045	1.008	0.941 to 1.079	1.017	0.946 to 1.094	1.011	0.95 to 1.075	0.996	0.912 to 1.087
BMI	0.998	0.995 to 1.000	0.999	0.994 to 1.004	0.998	0.993 to 1.004	0.997	0.992 to 1.001	0.996	0.989 to 1.002
Depression	0.974	0.937 to 1.012	1.000	0.928 to 1.079	0.983	0.908 to 1.064	0.949	0.887 to 1.015	0.966	0.877 to 1.062
**ANTIDIABETIC MEDICATION TYPE**										
**No diabetes medication**	**(ref)**	**(ref)**	**(ref)**	**(ref)**	**(ref)**	**(ref)**	**(ref)**	**(ref)**	**(ref)**	**(ref)**
Insulin and non-insulin	1.113	1.041 to 1.189	1.064	0.945 to 1.198	1.093	0.954 to 1.253	1.159	1.018 to 1.319	1.140	0.962 to 1.351
Insulin only	1.118	1.023 to 1.221	1.051	0.888 to 1.244	1.117	0.931 to 1.339	1.150	0.97 to 1.365	1.172	0.957 to 1.437
Non-insulin only	1.023	0.962 to 1.087	1.003	0.899 to 1.119	1.024	0.905 to 1.158	1.028	0.911 to 1.16	1.044	0.892 to 1.222

Gagnon J, Lussier M-T, MacGibbon B, Daskalopoulou SS and Bartlett G (2018) The Impact of Antidepressant Therapy on Glycemic Control in Canadian Primary Care Patients With Diabetes Mellitus. Front. Nutr. 5:47. doi: 10.3389/fnut.2018.00047
