# Supplementary material for: The Impact of Antidepressant Therapy on Glycemic Control in Canadian Primary Care Patients With Diabetes Mellitus
Source: Front Nutr. 2018 Jun 12;5:47. doi: 10.3389/fnut.2018.00047 (PMC6005871; doi:10.3389/fnut.2018.00047)
Supplement: Supplementary file 4 [file Table_4.pdf]

SUPPLEMENTARY TABLE 4 | Model predicting the association between antidepressants and change in HbA1c in diabetics with a history of depression, adjusting for body mass index

	Full model n=404		0 to 3 months n=227		3 to 6 months n=196		6 to 12 months n=194		12 to 18 months n=117	
	**Mean HbA1c ratio**	**95% CI**	**Mean HbA1c ratio**	**95% CI**	**Mean HbA1c ratio**	**95% CI**	**Mean HbA1c ratio**	**95% CI**	**Mean HbA1c ratio**	**95% CI**
Baseline HbA1c	1.078	1.060 to 1.096	1.088	1.059 to 1.118	1.073	1.032 to 1.116	1.067	1.035 to 1.100	1.076	1.021 to 1.133
**ANTIDEPRESSANTS**										
**Citalopram**	**(ref)**	**(ref)**	**(ref)**	**(ref)**	**(ref)**	**(ref)**	**(ref)**	**(ref)**	**(ref)**	**(ref)**
Amitriptyline	1.008	0.935 to 1.087	1.027	0.888 to 1.188	1.038	0.890 to 1.209	0.984	0.860 to 1.126	0.977	0.798 to 1.195
Venlafaxine	0.991	0.926 to 1.061	1.008	0.879 to 1.156	0.983	0.855 to 1.129	0.979	0.861 to 1.114	0.992	0.848 to 1.160
Trazodone	0.981	0.898 to 1.073	1.014	0.852 to 1.206	0.986	0.809 to 1.201	0.982	0.847 to 1.137	0.919	0.705 to 1.199
Escitalopram	0.985	0.908 to 1.068	1.001	0.871 to 1.151	1.040	0.867 to 1.247	0.946	0.815 to 1.098	0.951	0.743 to 1.218
Exposure duration (days)	1.000	1.000 to 1.000	1.000	0.998 to 1.002	1.000	0.997 to 1.002	1.000	0.999 to 1.001	1.000	0.999 to 1.001
**CHARACTERISTICS**										
Age	1.000	0.998 to 1.002	1.000	0.996 to 1.004	1.000	0.996 to 1.004	0.999	0.995 to 1.003	0.999	0.994 to 1.005
Sex (Women)	1.003	0.953 to 1.056	1.017	0.922 to 1.121	0.989	0.888 to 1.101	1.017	0.926 to 1.118	0.975	0.854 to 1.112
BMI	1.000	0.996 to 1.004	1.000	0.993 to 1.008	1.001	0.993 to 1.009	0.999	0.992 to 1.006	0.999	0.989 to 1.009
**ANTIDIABETIC MEDICATION TYPE**										
**No diabetes medication**	**(ref)**	**(ref)**	**(ref)**	**(ref)**	**(ref)**	**(ref)**	**(ref)**	**(ref)**	**(ref)**	**(ref)**
Insulin and non-insulin	1.122	1.015 to 1.241	1.095	0.906 to 1.323	1.090	0.884 to 1.345	1.159	0.962 to 1.396	1.161	0.901 to 1.496
Insulin only	1.129	0.989 to 1.290	1.081	0.838 to 1.394	1.092	0.832 to 1.434	1.196	0.936 to 1.529	1.158	0.839 to 1.599
Non-insulin only	1.029	0.938 to 1.128	1.032	0.866 to 1.229	1.019	0.847 to 1.226	1.032	0.872 to 1.222	1.048	0.830 to 1.322
